# Cartilage status in FAI patients – results from the Danish Hip Arthroscopy Registry (DHAR)

**DOI:** 10.1051/sicotj/2017023

**Published:** 2017-06-14

**Authors:** Bent Lund, Torsten Grønbech Nielsen, Martin Lind

**Affiliations:** 1 Department of Orthopaedics, Horsens Regional Hospital 8700 Horsens Denmark; 2 Department of Orthopaedics, Aarhus University Hospital THG 8000 Aarhus C Denmark

**Keywords:** FAI, Cartilage, HAGOS, National Registry

## Abstract

*Introduction*: The femoroacetabular impingement (FAI) morphology is associated with specific cartilage lesions, which are suspected to be early stages of the osteoarthritic development, which can be the end result of FAI. The cartilage status of FAI afflicted hip joints at the time of arthroscopic management is not fully elucidated. This study from the Danish Hip Arthroscopy Registry (DHAR) will try to show data on the cartilage status from a large cohort. Data from a national registry potentially represent large amounts of population-based epidemiological information from multiple centres and surgeons. Therefore, outcome data might be more reliable for a specific surgical intervention.

*Methods*: This study includes patients operated for symptomatic FAI from January 2012 until December 31st 2013, with a minimum of two-year follow-up and being registered in DHAR. The extent of cartilage damage at the time of surgery is reported and the Patient Related Outcome Measures (PROM) outcome data are presented.

*Results*: Data from a total of 686 FAI procedures in 1082 patients from January 2012 until December 31st 2013 were extracted from DHAR. Cartilage injuries were found in 88% of cases, mainly on the acetabular side. Overall PROM including pain scores improved significantly from preoperative status to follow-up one and two years postoperatively. The Copenhagen Hip and Groin Outcome Score (HAGOS), Hip Sports Activity Scale (HSAS) and global hip function showed less improvements in patients with more severe acetabular cartilage injury.

*Discussion*: The majority of patients with femoroacetabular impingement (FAI) undergoing hip arthroscopy have significant cartilage changes at the time of surgery primarily at the acetabulum and to a lesser degree at the femoral head. During FAI surgery the majority of patients have cartilage debridement performed but rarely cartilage repair. The presence of severe cartilage injury at the time of arthroscopic FAI surgery results in reduced subjective outcome and hip function.

## Introduction

Several studies have demonstrated evidence supporting the theory that FAI is a strong risk factor for the development of secondary osteoarthritis of the hip [1–6]. FAI morphology is associated with specific cartilage lesions, which are suspected to be early stages of the osteoarthritic development. The cartilage status of FAI afflicted hip joints at the time of arthroscopic management is not fully elucidated. Similarly there is still limited knowledge on the impact of cartilage injury on outcome after arthroscopic management of FAI. A systematic review from 2013 by Hetaimish et al. states that although FAI can be managed successfully by arthroscopic techniques, the current knowledge of the clinical outcome after FAI surgery is reported with a generally low evidence level mainly as case studies and with a lack of consistency in outcome reporting [7]. Cartilage pathology in FAI is believed to be caused by the impaction of both acetabular and femoral head cartilage edges onto the cam and pincer deformities of the FAI affected hip joint. The cartilage injuries in FAI are characterized by specific changes [8]. Early cartilage changes present as a bulging cartilage surface at the chondrolabral junction (wave sign), further lesion development results in a full thickness delamination and flap formation. Later changes are full thickness defects in the affected areas [9].

Presently several surgical strategies exist for the management of FAI-related cartilage lesions. These range from debridement of bulging and loose cartilage flaps, suturing and gluing of cartilage flaps to different repair techniques for areas of cartilage loss [10–12]. The repair techniques described are microfracture, collagen scaffold enhanced microfracture and different chondrocyte transplantation methods [13, 14].

National cohort studies have proven very successful for large-scale investigations of clinical outcome after arthroplasty and ligament reconstruction [15–17]. Data from a national registry potentially represent large amounts of population-based epidemiological information from multiple centres and surgeons. Therefore, outcome data might be more reliable for a specific surgical intervention. Registry data are potentially more representative for a specific surgical treatment than case series that may contain biased data due to selection of patients and highly dedicated surgeons.

Hip arthroscopy procedures in Denmark have been registered in the Danish Hip Arthroscopy Registry (DHAR) since the beginning of 2012. This registry is a voluntary clinical registry for patients and surgeons, and all of the centres performing hip arthroscopic procedures in Denmark do report to the registry [18]. One of the main indications in the registry for hip arthroscopic surgery is currently femoroacetabular impingement (FAI). The DHAR contains data on cartilage and labral pathology, which enables investigations on both the spectrum of cartilage pathology in FAI patients and the impact of cartilage status on the clinical outcome of arthroscopic FAI management.

The purpose of the present study is to present the intraarticular cartilage pathology in FAI patients and the management strategies to these cartilage lesions based on data from a national cohort. Also we shall investigate the impact of cartilage status on the clinical outcome of arthroscopic FAI management.

## Methods

### Clinical registry

The Danish Hip Arthroscopy Registry (DHAR) was initiated in 2012. Surgeons as well as patients report data online in an ongoing prospective data registration. For this study we extracted pre- and perioperative data including PROMs from DHAR between January 2012 and December 31st 2015. The postoperative patient reported data for this study were extracted at one and two-year follow-up. The registry is approved by the Danish Health Authorities, J.nr. 2012-58-0006.

The patient submits the preoperative subjective scores at registration consisting of various PROM and pain levels. At one, two and five years postoperatively the patient will be notified to submit their PROM scores at follow-up. After surgery, the surgeon reports data from clinical examination, radiological characteristics and the perioperative data.

### Patients in the present study

This study includes patients operated for symptomatic FAI from January 2012 until December 31st 2013, with a minimum of two-year follow-up. DHAR uses the three definitions of FAI types as described by Leunig et al. [3]: cam type, a developed asphericity of the femoral head and widening of the femoral neck, pincer type, a developed global or focal overcoverage on the acetabular side and mixed type, a combination of cam and pincer. We excluded data from patients without cartilage data registration, patients who were diagnosed with other diseases than FAI, previous surgery in the related hip or incomplete data registrations, patients with hip dysplasia defined as centre-edge (CE) angle of less than 25° and patients with revision hip arthroscopy.

### Registry data

#### Surgical findings and procedures

The operative data reported are the surgical procedure times including traction times, labral tears, cartilage injury assessment and surgical technique characteristics such as anchor usage, depth of rim trimming and cam resections.

The definition of acetabular cartilage injury type used in DHAR was initially classified by Beck et al. [2], and was later modified and validated by Konan et al. [19]. We have chosen to use the classification originally described by Beck, which is based on the Outerbridge classification. For femoral cartilage injuries the International Cartilage Repair Society (ICRS)-scale is used which is purely based on the injury depth [20].

### Patient outcome measurements

All PROMs used in DHAR are validated self-assessment scores and evaluated as suitable for patients undergoing hip arthroscopy. The used PROM questionnaires are the Copenhagen Hip and Groin Outcome Score (HAGOS), the EQ-5D and the Hip Sports Activity Scale (HSAS). The pain score used is the numeric rating scale (NRS). The HAGOS consists of six subscales assessing symptoms, pain, function in daily living, function in sport and recreation, participation in physical activities and hip and/or groin-related quality of life, each scored separately [21, 22]. HAGOS is a questionnaire (37 questions in total) aimed for young to middle-aged adults undergoing nonsurgical treatment or hip arthroscopy, but also patients presenting with groin pain. The EQ-5D is used as a generic health-related quality of life instrument, which is translated and validated into many languages [23]. The HSAS is recommended as a reliable and valid activity measurement useful for patients with femoroacetabular impingement [24]. Pain levels are measured using the NRS pain scores at rest and after 15 min of walking. A global hip function score using visual analogue scoring (VAS) from 0 to 100 was used, with 100 points being optimal hip function.

### Statistical analysis

The Student *t*-test was used to analyse the differences between the preoperative and postoperative PROM values. *P*-values below 0.05 were considered statistically significant. Subgroup analysis was made between patients with acetabular cartilage injury Beck grade 0–2 and grade 3–4 regarding PROM values using Student’s *t*-test for comparisons of PROM values.

## Results

### Population characteristics

Data from a total of 686 FAI procedures in 1082 patients from January 2012 until December 31st 2013 were extracted from DHAR. We excluded 396 procedures due to our exclusion criteria. Forty-seven patients had bilateral procedures performed. Forty-eight percent of the patients were female and 52% male. The average age at the time of surgery was 38.5 years (range, 12–76 years). The majority of the patients were characterized as mixed type FAI (87.3%). Isolated pincer type morphology consisted of only 4.9% whereas 7.8% were characterized as isolated cam type morphology.

### Registration completeness

The completeness of PROM registrations preoperatively was 58% and the follow-up completeness at one and two years was 55% and 52%, respectively. There is an ongoing study that is looking at data completeness comparing data from DHAR to the National Registry for Hospital Procedures.

### Cartilage injury findings and surgical procedures

Cartilage injuries were found in 88% of cases and the degree of injury for both acetabulum and femoral head is shown in Table 1. Normal cartilage was only found in 1.5% and 24% of the acetabular and femoral head joint surfaces, respectively. More severe cartilage injury of Beck or ICRS grade 3–4 was found in 44 and 7% of the acetabular and femoral head joint surfaces, respectively. Surgery due to cartilage injury was reported in 523 procedures (76%), with the most commonly performed surgical intervention being debridement with shaver or radiofrequency ablation technique. (Table 2) Cartilage repair procedure in the form of microfracture was only performed in 7% of cases. Cartilage flap re-attachment was performed in less than 1% of cases.

**Table 1.** Degree of injury for acetabulum and femoral head.

	Patients	%
Acetabulum (Becks)		
Grade 0: normal cartilage	10	1.5
Grade 1: fibrillation	95	13.8
Grade 2: wave sign	281	41.0
Grade 3: cleavage tear between labrum and articular cartilage	210	30.6
Grade 4: exposed bone in the acetabulum	90	13.1
Cartilage injury ≥ Grade 2	581	84.7
Femoral head (ICRS)		
Grade 0: normal cartilage	521	75.9
Grade 1: nearly normal	52	7.6
Grade 2: abnormal	68	9.9
Grade 3: partial loss of cartilage	29	4.2
Grade 4: exposed bone	16	2.3
Cartilage injury ≥ Grade 2	113	16.5
Combined cartilage injury		
Acetabulum *OR* femoral head cartilage injury ≥ Grade 2	601	88.2
Acetabulum *AND* femoral head cartilage injury ≥ Grade 2	93	13.6

**Table 2.** Cartilage procedures in patients undergoing arthroscopic FAI surgery.

Surgical procedure	Patients	%
Cartilage debridement any location	560	81.6
Cartilage reattachment femoral head	1	0.1
Cartilage reattachment acetabulum	4	0.6
Microfracture femoral head	3	0.4
Microfracture acetabulum	43	6.3

### Patient related outcome measures (PROM)

Overall PROM including pain scores improved significantly from preoperative status to follow-up one and two years postoperatively (Tables 3 and 4). We did not find any significant improvement in these parameters between one and two-year follow-up, except the NRS pain score for walking (*p* = 0.002). HAGOS demonstrated significant improvements in all subscales between the preoperative and the postoperative scores at one-year follow-up.

**Table 3.** Influence of cartilage injury on patient reported outcome in patients undergoing arthroscopic FAI surgery.

	1 year data				2 year data			
	Grade 0–2		Grade 3–4		Grade 0–2		Grade 3–4	
	Mean	*SD*	Mean	*SD*	Mean	*SD*	Mean	*SD*
HAGOS								
Pain	73	23	70	21	74	22	72	20
Symptoms	68	21	66	19	69	21	68	19
ADL	75	24	73	24	77	25	74	23
Sport	61	30	56	27	64	29	55*	28
PA	48	35	39*	34	51	35	40*	36
QOL	55	27	50	26	59	26	51*	26
NRS – rest	19	22	19	21	19	24	16	18
NRS – activity	28	31	30	28	23	25	21	22
VAS	29	25	33	23	28	25	34*	25
EQ5D	0.78	0.19	0.76	0.17	0.79	0.19	0.77	0.16
HSAS	3.1	2.1	3.2	2.0	3.3	2.0	3.3	1.9

* Significant difference between grade 0–2 and grade 3–4, *p* < 0.05.

**Table 4.** Outcome differences between 2 years FU and baseline values.

	2 year data			
	Grade 0–2		Grade 3–4	
	Mean	*SD*	Mean	*SD*
HAGOS				
Pain	22	20	17	21
Symptoms	19	19	15	21
ADL	25	23	19	25
Sport	29	27	18*	30
PA	33	34	16*	40
QOL	29	25	17*	23
NRS – rest	20	28	18	26
NRS – activity	30	40	25	30
VAS	29	26	17*	30
EQ5D	0.14	0.18	0.20	0.20
HSAS	0.9	2.2	0.4*	1.8

* Significant difference between grade 0–2 and grade 3–4, *p* < 0.05.

The impact of having a significant acetabular cartilage injury of Beck grade 3–4 is presented in Tables 3 and 4. We found significantly less improvement of the following PROM parameters when more severe acetabular cartilage injury was present: HAGOS, HSAS and global hip function.

## Discussion

Key findings in the present study are that patients with symptomatic FAI in a large national cohort have significant cartilage changes at the time of surgery, mainly on the acetabular side. Data from the DHAR shows that 88% of the patients have cartilage changes and 97% of these were located at the acetabular side. Seventy-seven percent of patients undergo some kind of cartilage procedure during the surgical FAI management.

We also found that patients undergoing FAI surgery do benefit from hip arthroscopic procedures involving labral reinsertion, cartilage surgery and removal of cam and pincer bone deformities within a 2-year follow-up. We also found that patients with cartilage injury grade 3 to 4 have inferior results than those with no or minor cartilage injury.

A systematic review by Nwachukwu et al. compared open vs. arthroscopic FAI surgery and demonstrated excellent and comparable hip survival rates at medium to long-term follow-up. Hip arthroscopy showed a statistically significant improvement in general health-related quality of life, when compared to open procedures [25]. In the study with THA as an outcome endpoint, there was an overall survival rate of 93% for open and 90.5% for arthroscopic procedures. A recent retrospective population-based analysis (*n* = 7351) by Schairer et al. showed an overall conversion rate of 12.4% within two years of hip arthroscopic surgery [26]. The lowest rate of conversion was found in the age group younger than 40 years, at 3%. The highest risk of conversion was seen in the age group 60 to 69 years, at 35%.

A systematic review by Weber et al. described a rate of reoperations after hip arthroscopy at 6.3% in a cohort consisting of more than 6000 from 92 studies [27]. The majority of these reoperations were, respectively, revision hip arthroscopy in 1.9% and conversion to THA in 2.9% of the patients.

In a recently published study by Malviya et al. of more than 6000 hip arthroscopies registered between 2005 and 2013 in the British National Health Service, a revision hip arthroscopy rate of 4.5% was found [28]. Their conversion rate to THR was 10.6%. In the present study, revision hip arthroscopy was performed in 7.3% of cases and conversion to THA was reported in 1.6% of the cases.

We found that a large proportion (88%) of this FAI cohort had chondral injuries at the time of surgery. The most common site of cartilage injury was in the acetabulum and to a minor degree on the femoral head. Although many of these injuries were described as full thickness cartilage damage, a repair procedure was not frequently performed. In the DHAR, debridement and radiofrequency ablation of cartilage injuries were the most common procedures compromising more than 91% of all cartilage procedures.

In a large cross-sectional study of a North American FAI cohort, the most common surgical technique during FAI surgery was femoral head-neck osteochondroplasty in 91.6% of the 1130 procedures. However, CAM deformity resection was not considered a cartilage procedure in the DHAR. The percentage of acetabular microfracture interventions in this study was similar to the reported number in DHAR, respectively, 5.6% and 5.4% [29].

Several cartilage repair strategies have been suggested for management of the cartilage lesion found during hip arthroscopy in FAI patients. Early changes can be managed by the debridement of bulging and loose cartilage flaps. More advanced methods are suturing and gluing of cartilage flaps to different repair techniques for areas of cartilage loss [10–12]. The repair techniques described are microfracture, collagen scaffold enhanced microfracture and different chondrocyte transplantation methods [13, 30, 31]. Microfracture as described by Steadman et al. [32] is a readily available cartilage repair technique for hip arthroscopy. The efficacy for hip cartilage repair has still not been investigated. But experience from the knee has shown that microfracture can form fibrocartilage that can result in symptom relief in 70% of patients [33]. One single case series of 24 FAI patients with microfracture treatment of cartilage lesions and second-look arthroscopic evaluation of healing found excellent filling in 95% patients. Histologic evaluation of repair tissue from two patients demonstrated fibrocartilage tissue characteristics [34].

Microfracture repair can be enhanced with the application of a collagen scaffold on top of the defect after microfracture using the so-called autologous matrix-induced chondrogenesis (AMIC) technique. This technique was compared with chondrocyte transplantation by Mancini and Fontana describing good outcomes long-term with both techniques resulting in 38–39 point improvements in modified Harris hip score without any difference between techniques [13].

One major problem for investigations of the impact of cartilage procedures in FAI surgical management is that the cartilage procedure is secondary to procedures done to remove the FAI pathology such as removal of cam and pincer deformities and labral repairs. A recent systematic review on cartilage injury management by Marquez-Lara et al. states that only level IV and V studies are present and that there is a need for good level I studies [14].

The present study and studies previously mentioned show consistently improved clinical outcomes of hip arthroscopy in the treatment of FAI and the concurrent labral damage, especially regarding patient’s quality of life and pain-related outcome measures. However, the impact of cartilage debridement and repair is poorly investigated and there is a major challenge scientifically since the cartilage procedures are secondary to the FAI procedures. There is a need for good level I studies comparing different surgical treatment strategies of FAI with and without cartilage management.

### Limitations

There are limitations in the present study with potential data quality issues due to the potential diversity in interpretation of the intraarticular pathology findings done by the individual surgeons and due to completeness of data in the Danish Hip Arthroscopy Registry. All data input is voluntary, both for surgeons and patients. We know from the Danish National ACL Registry that patient inputs are as low as 35% but more than 85% from surgeons [35]. The hip arthroscopy procedure is regulated by the Danish Board of Health and is therefore limited to the 11 centres with permission to perform the procedure. The actual number of hip arthroscopic procedures during this time period in Denmark is not known, but is being studied in an ongoing validation study. Further studies based on central healthcare registries are needed to document the degree of completeness for surgical data. Finally, DHAR does not include data from patients undergoing nonsurgical treatments of FAI, which would be of interest for outcome studies. However, the DHAR utilizes prospective data collection without potential bias from future study purposes such as the present study. The high data volume and the diversity of clinics and surgeons potentially provide data that to a high degree reflects the true pathology status and outcome profile of FAI patients.

## Conclusion

The majority of patients with femoroacetabular impingement undergoing hip arthroscopy have significant cartilage changes at the time of surgery primarily at the acetabulum and to a lesser degree at the femoral head. During FAI surgery the majority of patients have cartilage debridement performed but rarely cartilage repair. The presence of severe cartilage injury at the time of arthroscopic FAI surgery results in reduced subjective outcome and hip function.

## Conflict of interest

The authors declare no conflict of interest in relation with this paper.
